# Composition and diversity of gut microbiota in diabetic retinopathy

**DOI:** 10.3389/fmicb.2022.926926

**Published:** 2022-08-23

**Authors:** Jianhao Bai, Zhongqi Wan, Yuanyuan Zhang, Tianyu Wang, Yawen Xue, Qing Peng

**Affiliations:** Department of Ophthalmology, Shanghai Tenth People’s Hospital of Tongji University, Tongji University School of Medicine, Shanghai, China

**Keywords:** diabetic retinopathy, gut microbiota, human, biomarker, 16S rRNA gene amplicon sequencing

## Abstract

**Objective:**

Diabetic retinopathy (DR) is one of the most common complications of type 2 diabetes mellitus. The current study investigates the composition, structure, and function of gut microbiota in DR patients and explores the correlation between gut microbiota and clinical characteristics of DR.

**Methods:**

A total of 50 stool samples were collected from 50 participants, including 25 DR patients and 25 healthy controls (HCs). 16S ribosomal RNA gene sequencing was used to analyze the gut microbial composition in these two groups. DNA was extracted from the fecal samples using the MiSeq platform.

**Results:**

The microbial structure and composition of DR patients were different from that of HCs. The microbial richness of gut microbiota in DR was higher than that of normal individuals. The alterations of microbiome of DR patients were associated with disrupted Firmicutes, Bacteroidetes, Synergistota, and Desulfobacterota phyla. In addition, increased levels of *Bacteroides*, *Megamonas*, *Ruminococcus_torques*_group, *Lachnoclostridium*, and *Alistipes*, and decreased levels of *Blautia*, *Eubacterium_ hallii*_group, *Collinsella*, *Dorea*, *Romboutsia*, *Anaerostipes*, and *Fusicatenibacter* genera were observed in the DR groups. Additionally, a stochastic forest model was developed to identify a set of biomarkers with seven bacterial genera that can differentiate patients with DR from those HC. The microbial communities exhibited varied functions in these two groups because of the alterations of the above-mentioned bacterial genera.

**Conclusion:**

The altered composition and function of gut microbiota in DR patients indicated that gut microbiome could be used as non-invasive biomarkers, improve clinical diagnostic methods, and identify putative therapeutic targets for DR.

## Introduction

A large number of bacteria colonize human guts. The total number of bacteria in the adult intestine is 10 times greater than the number of human cells (Shivaji, 2017). These microorganisms, weighing approximately 1.13 kg, have a highly co-evolutionary relationship with the mammalian immune system and interact with the host environment, participating in a variety of physiological processes, such as maintaining the immune system balance, energy homeostasis, nutrient production, anti-inflammatory responses, and signaling pathway modulation (Pflughoeft and Versalovic, 2012). Accumulating evidence shows a profound association between dysbiosis in the gut microbiota and the pathogenesis of several human systemic diseases, including inflammatory bowel disease (Halfvarson et al., 2017), irritable bowel syndrome (Bernstein and Forbes, 2017), Alzheimer’s disease (Wang et al., 2019), Parkinson’s disease (Caputi and Giron, 2018), and depression (Zheng et al., 2020). This phenomenon is further substantiated by the theory of gut microbiota–brain axis (Caputi and Giron, 2018; Angelucci et al., 2019; Wang et al., 2019; Morais et al., 2021). Stabilizing the gut–retina axis is effective in treating ocular disorders. Several studies have highlighted that both the gut microbiota and its metabolites might affect ocular immunity through molecular simulation and comprehensive immune pathway, which could proceed to destroy tissues throughout the body and lead to the onset of ocular diseases. One speculation is that in a certain disease circumstance, gut microbiota and its metabolites may destroy the mucosal barrier and increase intestinal permeability, and temporary breach of blood–retina barrier gives them a chance to enter the eye, leading to the occurrence and development of ocular diseases via stimulation of both the innate and adaptive response of the immune system.

The discovery that gut microbiota modulates retinal function has increased the awareness of the link between gut microbiome and ophthalmic disease (Rowan et al., 2017; Rowan and Taylor, 2018). Based on the next-generation sequencing, recent decades have seen ample evidence documenting dysbiosis in the gut microbiota has been linked to multiple ocular disorders. For example, dysbiosis in gut bacterial communities has been observed in recipient experimental autoimmune uveitis mice and uveitis patients (Janowitz et al., 2019). Zinkernagel had revealed that intestinal dysbiosis, such as the bacterial genera Anaerotruncus and Oscillibacter and the species Ruminococcus torques and *Eubacterium ventriosum*, might occur in individuals with age-related macular degeneration (Zinkernagel et al., 2017). Gong also demonstrated that patients with glaucoma have imbalance of gut microbiota and distinct profiles of serum metabolites compared to healthy adults (Gong et al., 2020). In addition, the impact of microbiota on eye diseases has also been observed. However, the association between the pathological process of diabetic retinopathy (DR) and human gut microbial community is yet unclear.

Diabetic retinopathy is a major complication of diabetes and the cause of vision loss and blindness. Impaired vision could be attributed to macular edema and ischemia, vitreous hemorrhage, retinal detachment, neovascularization of the retina and iris, and neovascular glaucoma (Cheung et al., 2010). Vascular endothelial growth factor (VEGF) is now known to play a central role in the development and progression of DR, and anti-VEGF therapy were found to be highly sufficient to improve the prognosis of visual acuity in patients with DR, but not all patients can benefit from it. For these patients, researchers hope to find new pathogenic molecules and alternative therapies. The etiology of DR is multifactorial, diet and nutrition have a profound epidemiologic association with the occurrence and development of diabetes and its complications. Therefore, further understanding of the link of gut microbiota and DR might provide new clinical strategies against the disease.

The composition of gut microbiota alters during the development of diabetes. Standardized fecal bacterial transplantation decreases insulin resistance and improves insulin sensitivity, thus arresting the progress of type 2 diabetes mellitus (T2DM) (de Groot et al., 2021).

Although previous studies indicated the alterations of gut microbiota on diabetes mellitus in detail (Qin et al., 2012; Vatanen et al., 2018), the correlation between human gut microbiota and DR has not yet been elucidated. Recent advances indicate a critical role of gut microbiota in the onset and progression of DR in mouse models; for example, alterations in the gut microbial composition and function via intermittent fasting prevent retinopathy and prolong the survival of mice, suggesting the participation of gut bacteria in the onset and progression of DR (Beli et al., 2018). Interestingly, the gut bacterial composition and function of diabetic patients with or without retinopathy might be variable. Hitherto, a few studies have explored the functional and metabolic changes in gut microbial communities among DR patients using high-throughput gene sequencing techniques.

In this study, we collected fecal samples from the DR patients and healthy controls (HCs) and delineated the community structure of the gut microbiome of DR patients based on 16S ribosomal RNA (rRNA) gene sequencing. In addition, the differences in the composition and function of the gut microbiota between patients with DR and HCs were investigated systematically. We aimed to develop a non-invasive method for diagnosing and identifying potential microbial targets to improve our understanding of pathogenesis and therapeutic approaches for DR.

## Materials and methods

### Protocol approvals and patient consent

This clinical study was approved by the ethics review committee of Shanghai Tenth People’s Hospital, Shanghai, China (Ethics Approve No. SHSY-IEC-5.0/22K100/01) and registered in the China Clinical Trials Registration Center (No. ChiCTR2200058763).^1^ The protocol in this study conformed to the Declaration of Helsinki. Informed consents were obtained from all the subjects.

### Recruitment of the study subjects

A total of 25 DR patients and 25 age- and sex-matched HCs were enrolled in this study. The participants included resident men and women (Shanghai, China). They were examined by ultra-wide-field scanning laser ophthalmoscopy (UWF-SLO) and optical coherence tomography angiography (OCTA). In addition, fundus fluorescein angiography (FFA) and indocyanine green angiography (ICGA) were conducted in all DR patients. The inclusion criteria of the experimental group were as follows: (1) age 25–70 years; (2) fulfilling the 1999 World Health Organization (WHO) diagnostic criteria for T2DM; (3) DR was diagnosed based on the International Clinical Diabetic Retinopathy Disease Severity Scale (2002). Regardless of the level of severity, patients with retinopathy were classified into the DR group; (4) provided informed consent; (5) did not have any other intraocular diseases. The spouses of patients with DR were recruited as the HC group, all the participants have no obvious dietary differences from their spouses, they had no ocular diseases other than refractive errors, no diabetes, and no other major systemic diseases. In addition, the difference of age between the healthy individuals and their spouses was ≤3 years.

The exclusion criteria were as follows: (1) usage of antibiotics within 6 weeks before participation in the study; (2) usage of probiotics and prebiotics within 3 months prior to fecal sampling.; (3) history of abdominal surgeries; (4) daily alcohol consumption >30 g; (5) significant immunodeficiency, serious kidney or liver disease; (6) breastfeeding or pregnancy.

In addition, we confirmed all subjects had relatively similar diet structures using a standardized 4-week recall questionnaire, which was discussed with a dietician.

### Sample collection and processing

Body mass index (BMI) was calculated at the beginning of the study. The fecal samples were collected in cold, sterile stool containers, transported to the laboratory within 2 h of collection, and stored at −80°C until microbiota analysis.

### DNA extraction and polymerase chain reaction amplification

Total microbial DNA was extracted from frozen fecal samples using E.Z.N.A.^®^ Soil DNA Kit (Omega Bio-Tek Inc., United States) according to the manufacturer’s protocol. The quality and concentration of DNA were determined on 1% agarose gel electrophoresis and a NanoDrop^®^ ND-2000 spectrophotometer (Thermo Scientific Inc., United States), respectively. The hypervariable region V3–V4 of the bacterial *16S* rRNA gene was selected as the target for amplification with primer sequences 338F (5′-ACTCCTACGGGAGGCAGCAG-3′) and 806R (5′-GGACTACHVGGGTWTCTAAT-3′) on an ABI GeneAmp^®^ 9700 PCR thermocycler (ABI, CA, United States). The PCR reactions were performed using TransStart Fast Pfu DNA Polymerase (TransGen, Beijing, China) in a 20 μL reaction, consisting of 2 μL of 2.5 mM dNTPs, 0.4 μL Fast Pfu polymerase, 0.8 μL each primer (5 μM), 4 μL of 5× Fast Pfu buffer, and 10 ng of template DNA. The AxyPrep DNA Gel Extraction Kit (AXYGEN Biosciences, Union City, CA, United States) was used to purify the amplicon products, and all samples were amplified in triplicate. Then, the purified amplicon products were pooled in equimolar amounts, followed by paired-end sequencing on an Illumina MiSeq platform (Illumina, San Diego, CA, United States) according to the manufacturer’s instructions (Majorbio Biopharm Technology Co., Ltd., Shanghai, China).

### Data processing

Raw FASTQ files were demultiplexed, quality-filtered, and merged using the following steps. First, to obtain clean data, the 300-bp reads were truncated at any site if the average quality score was <20, with a 50-bp sliding window, and reads with quality scores <50 were discarded. Second, only overlaps >10 bp were assembled based on their overlapped sequence, with the maximum mismatch ratio set to 0.2. Third, the samples were distinguished based on the barcode/primers and barcode matching, followed by adjustment of the sequence direction. Uparse v7.1^2^ was used to cluster the optimized sequences into operational taxonomic units (OTUs) with 97% sequence similarity cutoffs. Then, the most abundant sequence was selected as the representative sequence. Due to the individual differences among various samples, the sequences of all samples were randomly selected into a uniform data body according to the lowest sequence number to compare each sample at the same OTU serial number level. Phylogenetic Investigation of Communities by Reconstruction of Unobserved States (PICRUSt2) was used to predict the metagenomic function.

### Statistical analysis

Based on the OTU information, a-diversity was evaluated by the species indices (Sobs, Chao, Shannon, Invsimpson, Shannoneven, and Heip), calculated using Mothur v1.30.1, and β-diversity was used to explore the diversity of the microbial structure. Principal coordinate analysis (PCoA) and partial least squares discriminant analysis (PLS-DA) was performed to determine the similarity among the microbial communities in various samples using Vegan v2.5-3 package. The linear discriminant analysis (LDA) effect size (LEfSe) was used to identify the differences in taxa between DR and HCs (phylum to genera). In this method, a non-parametric factorial Wilcoxon rank-sum test was performed to detect the features with significant differential abundance, and the effect size of each feature using LDA was calculated. Statistical analyses were performed with R software (v.3.4.1). Statistical analyses were performed using SPSS v25 (IBM, United States). *P*-value <0.05 indicated statistical significance.

## Results

### Clinical characteristics

**FIGURE 1:**
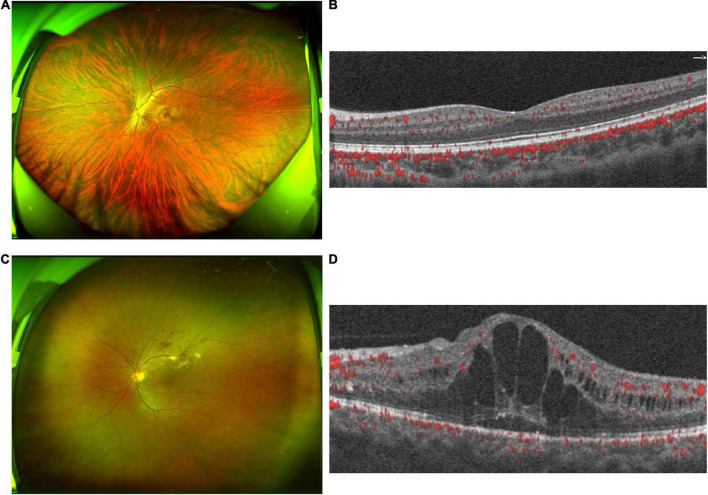
Clinical features of diabetic retinopathy (DR) patients and healthy controls (HC). **(A)** The ultra-wide-field fundus of HCs showed a normal structure of fundus. **(B)** The optical coherence tomography angiography (OCTA) images of HCs showed a normal structure of fundus. **(C)** The ultra-wide-field fundus ophthalmoscopy in the DR group showed retinal vascular lesions, including microaneurysms, hemorrhages, cotton wool spots, and lipid exudates. **(D)** OCTA images in the DR group show macular edema and retinal thickening. OCTA, optical coherence tomography angiography.

A total of 50 participants, including 25 DR patients and their healthy spouses, were recruited in this study. Three samples from healthy subjects were excluded due to insufficient size or inadequate PCR amplification. The researchers have tried PCR amplification several times, but these samples did not meet the requirements and hence, 47 stool samples were tested. All patients with DR were diagnosed with T2DM for >10 years; the longest duration was >30 years. No statistically significant differences were detected in age and BMI between the two groups (*P* > 0.05, Table 1). The ultra-wide-field fundus and OCTA images of HCs showed a normal structure (Figures 1A,B). However, the fundus images of DR patients showed various retinal vascular lesions, including microaneurysms, punctate hemorrhages, lipid exudates, and cotton wool spots (Figure 1C). The OCTA images in the DR group showed significant macular edema and retinal thickening (Figure 1D).

**TABLE 1:** Characteristics of the two groups.

	DR (*n* = 25)	HC (*n* = 22)	*P-value*
Age (year)	55.64 ± 6.10	56.32 ± 6.56	0.721
Male (n,%)	13 (52%)	11 (50%)	0.891
BMI (kg/m^2^)	24.11 ± 3.01	23.73 ± 2.85	0.666
History of smoking (n,%)	5 (20%)	3 (13.6%)	0.849
History of drinking (n,%)	3 (12%)	2 (9.1%)	1.000
Hypertension (n,%)	12 (48%)	13 (59.1%)	0.447
Diabetes (n,%)	25 (100%)	0 (0%)	<0.001*

BMI, body mass index. The * means p < 0.05.

### Diabetic retinopathy significantly altered the gut microbiota diversity

Next-generation sequencing of the V4 high-variable regions of the *16S* rRNA gene was used to detect and identify the bacterial profiles in the fecal microbiota using the Illumina MiSeq sequencing platform. The total number of high-quality sequences obtained for 47 subjects was 2,193,685, with an average length of 411. The sequencing results of each sample are presented in Supplementary Table 1. Based on the minimum number of sequences per sample, these sequences were distinguished, filtered, and clustered into 807 OTUs at 97% sequence similarity.

**FIGURE 2:**
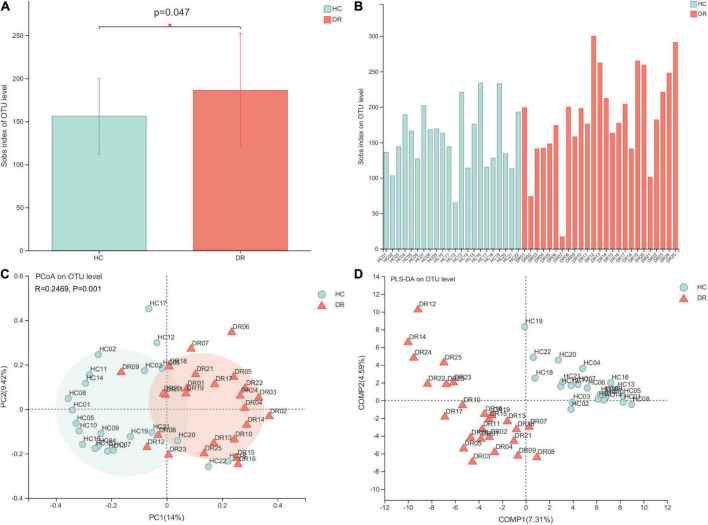
Comparison of alpha diversity and beta diversity in patients with diabetic retinopathy (DR) and healthy controls (HCs). **(A)** Sobs alpha diversity index is significantly increased in DR compared to HC individuals (*P* = 0.047 Wilcoxon rank-sum test). **(B)** Sobs alpha diversity index of each fecal sample. **(C)** Principal coordinate analysis (PCoA) plot at the operational taxonomic unit (OTU) level. **(D)** Partial least squares discriminant analysis (PLS-DA) showed an obvious distinction at the OTU level between the microbiota composition of the two groups. *P < 0.05.

Alpha-diversity refers to species diversity within communities. In the current study, alpha diversity indices, including species richness indices (Sobs and Chao), species evenness indices (Shannoneven and Heip), and species diversity indices (Shannon and Invsimpson), were used to test the index values between the two groups. We found significant difference in species richness indices (sobs index) between the two groups (*P* < 0.05), indicating that the microbial richness of gut microbiota in patients with DR was higher than that of normal individuals (Figure 2A). The sobs index on the OTUs for each sample is shown in Figure 2B. However, the other indices did not reach statistical significance between the DR and HC groups. In addition, beta diversity was analyzed to assess the overall difference and similarity in the structure of microbial population among the groups. An obvious distinction was observed between the microbiota composition of the two groups based on Bray–Curtis distances, as shown in the PCoA plot at the OTU level (*P* = 0.001, Figure 2C). In addition, PLS-DA also distinguished the groups at the OTU level (Figure 2D). The PLS-DA distance revealed that the bacterial microbiome composition of DR patients was different from that of HC subjects. Overall, the comparison of alpha diversity and beta diversity in the DR and HC groups indicated that patients with DR harbored an altered bacterial gut microbiome.

### Differential bacterial composition and abundance in diabetic retinopathy and healthy control groups

**FIGURE 3:**
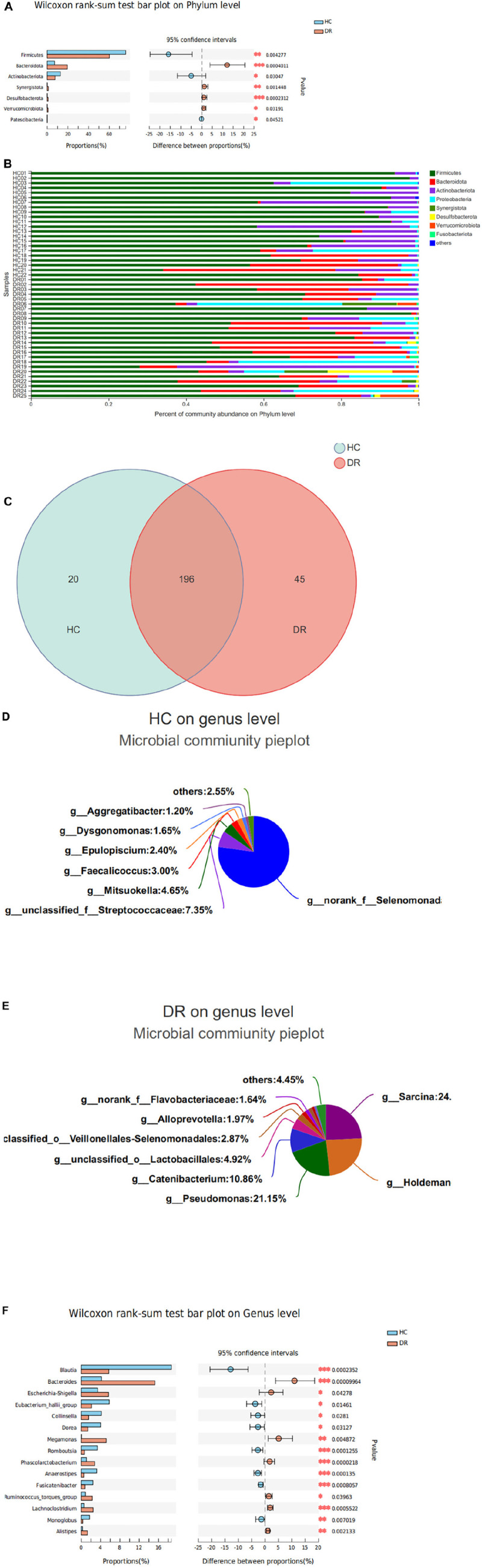
Variations in fecal microbiota composition at the phyla and genus level in patients with diabetic retinopathy (DR) and healthy controls (HCs). **(A)** Three phyla were enriched in the HC group; four families were enriched in the DR group. **(B)** Column chart showed the same dominant species of different samples at the phylum level, but the relative abundance is different. **(C)** The pie chart showed the overlapped and unique genera in the two groups. Most of the genera (196) overlapped and were observed in all groups. **(D)** A total of 20 genera detected only in the HC group. **(E)** A total of 45 genera detected only in the DR group. **(F)** Variations in fecal microbiota composition at the genus level. *P < 0.05, ^**^P < 0.01, ^***^P < 0.001.

The relative abundance of microbial composition in the two groups was compared at the phylum and genus levels. Herein, we identified 15 phyla and 274 genera in all the samples. According to the clustering results, most of the sequences of the subjects in this study belonged to the five most abundant bacterial phyla: Firmicutes, Bacteroidota, Actinobacteriota, Proteobacteria, and Verrucomicrobiota. Consistent with our findings, previous studies have reported that these phyla constitute the majority of the human intestinal microbiota (De Filippo et al., 2010). Overall, the comparison between the relative abundance of each phylum in the two groups revealed that Firmicutes was less abundant in the DR group than in the HC group. In addition, Bacteroidota, Synergistota, Desulfobacterota, and Verrucomicrobiota were more abundant in the DR group than in the HCs (*P* < 0.05; Wilcoxon rank-sum test; Figure 3A). The results of the column chart revealed that the dominant species of various samples are identical at the phylum level, but the relative abundance is different (Figure 3B).

A total of 274 genera were identified. In all fecal samples, *Blautia*, *Bacteroides*, *Bifidobacterium*, *Eubacterium_hallii*_group, and *Escherichia-shigella* were the top five species at the genus level. Furthermore, 33 major genera were distributed in the two groups with relative abundance. Among these, *Bacteroides* was the most abundant genus in the DR group, while *Blautia* was the most abundant genus in the HC group. Most of the genera (196) overlapped and were observed in all groups. Of the 20 genera detected only in the HC group, 84.55% was the no-rank_f_Selenomonadac and un-classified_f_Streptococcaceae genera. A total of 45 genera were detected in the DR group, with 84.15% belonging to *Sarcina*, *Holdeman*, *Pseudomonas*, and *Catenibacterium* (Figures 3C–E). Also, a higher abundance of *Bacteroides*, *Megamonas*, *Ruminococcus_torques*_group, *Lachnoclostridium*, and *Alistipes* and lower abundance of *Blautia*, *Eubacterium_hallii*_group, *Collinsella*, *Dorea, Romboutsia*, *Anaerostipes*, and *Fusicatenibacter* genera was observed in the DR group than in the HC group (Figure 3F).

**FIGURE 4:**
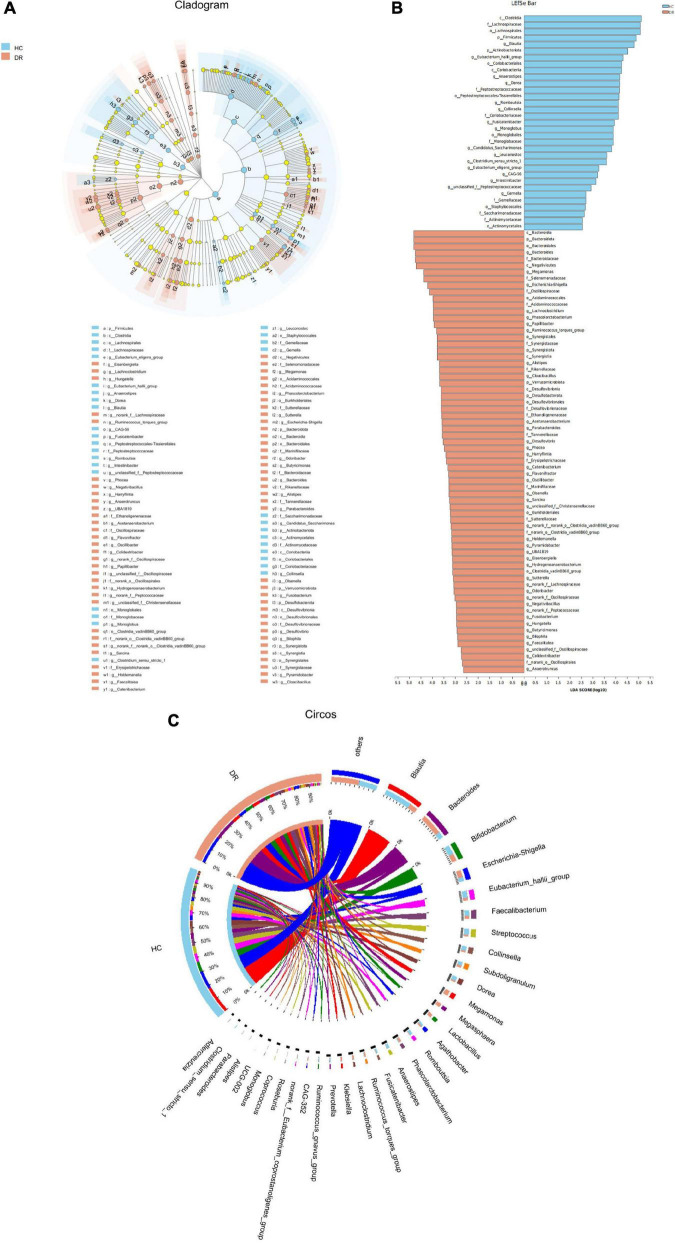
Relative abundance of the bacterial community in patients with diabetic retinopathy (DR) and healthy controls (HCs). **(A)** LEfSe analysis of the fecal microbiota composition from the phylum to the genus level in the two groups. The cladogram displayed the correlations between taxa at different taxonomic levels. Each circle represents a hierarchy, followed by phylum, class, order, family, and genus. Different phyla were marked with different colors. The size of the nodes represents the taxon abundance. **(B)** LEfSe analysis showed the relative abundance of genera in the DR and control groups. A total of 101 bacterial taxa showed significant differences in relative abundance, with 33 and 68 distinct microbial taxa in the HC and DR groups, respectively (LDA score >2.5, *P* < 0.05, Kruskal–Wallis test). **(C)** Circos sample and species diagram reflected the correlation between samples and species. LEfSe, linear discriminant analysis effect size; LDA, linear discriminant analysis.

To further analyze and characterize the distinct bacterial community structure in the DR and control groups, we performed LFfSe analysis (an algorithm for high-dimensional biomarker discovery) on the fecal microbiota composition from the phylum to the genus level using LDA to estimate the effect size of each taxon that were differentially represented in the two groups. Additionally, 101 bacterial taxa showed significant differences in the relative abundance in DR and HC groups, with 68 distinct microbial taxa in the DR group. These taxa belonged to 25 main families (LDA score >2.5, Kruskal–Wallis test, *P* < 0.05; Figures 4A,B). Firmicutes, Bacteroidetes, Synergistota, and Desulfobacterota phyla in the DR group and dysbiosis of the gut microbiome from the phylum to the genus level in the DR group were observed.

In order to intuitively describe the correlation between samples and species, Circos sample and species diagram were used to reflect the proportion of composition of dominant species in each group and the distribution of dominant species in various groups (Figure 4C). The results of the Circos sample and species diagram were consistent.

### Gut microbial biomarkers for discriminating diabetic retinopathy and healthy control

Bacterial genera were analyzed using a random forest classifier to identify the microbial characteristics that can distinguish between the DR and HC groups. Based on the model evaluation, we selected the most important feature with the highest value of area under the curve (AUC) as the biomarker to distinguish the two groups.

**FIGURE 5:**
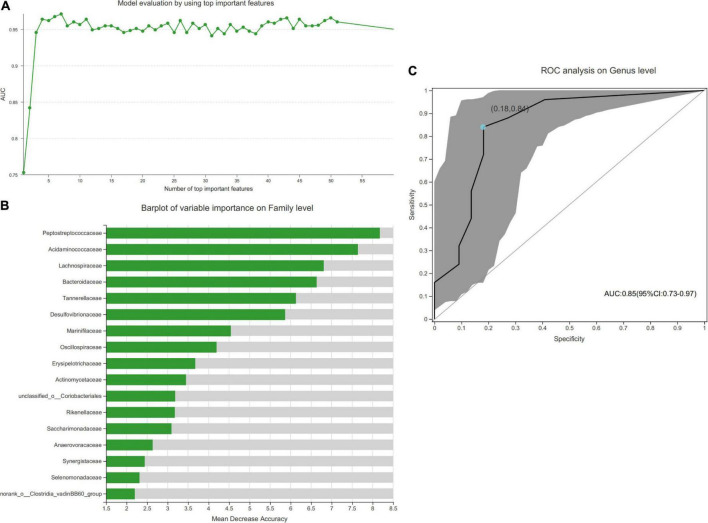
Disease classification based on gut microbial biomarkers. **(A)** The AUC-RF algorithm is used to determine a stochastic forest optimal model to maximize the area under the curve (AUC) value of the receiver operating characteristic (ROC) curve, and the number of species selected for the importance ranking is 7 when the highest AUC value is 0.9709. **(B)** The results of sorting 17 important species. **(C)** Classification performance of the multivariable logistic regression model using the combination of *Blautia*, *Bacteroides*, *Megamonas*, *Romboutsia*, and *Anaerostipes* was assessed based on the AUC (0.85).

The AUC-RF algorithm was used to determine a stochastic forest optimal model to maximize the AUC value of the receiver operating characteristic (ROC) curve. At the highest AUC value (0.97), 7 species were ranked according to importance (Figure 5A). Figure 5B shows the results of the top 17 species. Then, to assess the potential value of the gut microbiota model as a biomarker, we applied ROC curve analysis to quantify their classification ability based on the AUC value. Also, the potential value of *Blautia*, *Bacteroides*, *Megamonas*, *Romboutsia*, and *Anaerostipes* as biomarkers was assessed. We found that the combination of these microbial biomarkers could discriminate DR patients from the HC group with an AUC of 0.85 (95% CI: 0.73–0.97) (Figure 5C).

### Microbial functional alteration

**FIGURE 6:**
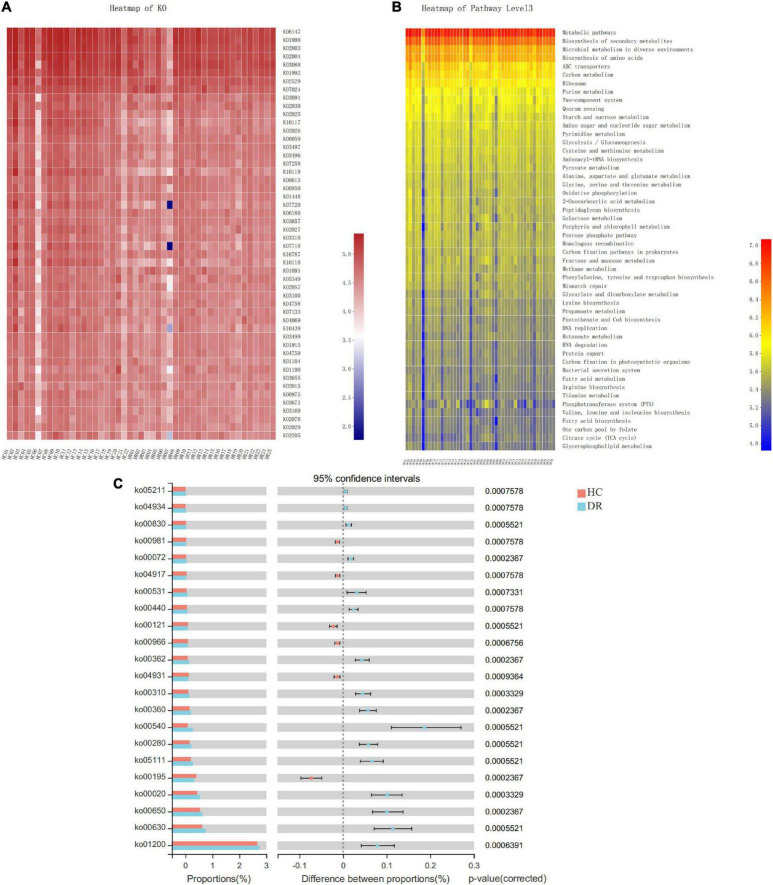
Functional analysis of the predicted metagenomes. **(A)** Heatmap of KEGG ortholog (KO) genes among the two groups. **(B)** Heatmap of Kyoto Encyclopedia of Genes and Genomes (KEGG) function pathway at level 3. **(C)** The significantly a differential pathway at level 3 between diabetic retinopathy (DR) patients and healthy control (HC) individuals.

To examine the functional changes of the microbial communities in the DR and HC groups, we used PICRUSt2 to infer the metagenomes from the 16S rRNA data and predict the functional profile of the microbial community. PICRUSt2 is a software tool that provides a starting point for understanding the altered functions within a microbiota flora. In the current study, a total of 6507 Kyoto Encyclopedia of Genes and Genomes (KEGG) ortholog (KO) genes and 335 KEGG pathways from the entire data set were predicted (Supplementary Tables 2, 3). In agreement with a previous study (Du et al., 2021), we found that these pathways were mainly distributed in the metabolism and genetic information processing of KEGG. Then, we used a heatmap to depict and compare the KO genes and microbiota function pathway at level 3 in these two groups (Figures 6A,B). In addition, the pathways involved in “insulin resistance” and “glucosinolate biosynthesis” were suppressed in DR patients. The predicted KEGG pathways were differently related to other several metabolic pathways involved in carbohydrate metabolism [“glyoxylate and dicarboxylate metabolism” “butanoate metabolism” “citrate cycle (TCA cycle)”], glycan biosynthesis and metabolism (“lipopolysaccharide biosynthesis” and “glycosaminoglycan degradation”), metabolism of cofactors and vitamins (“phosphonate and phosphinate metabolism” and “retinol metabolism”), and amino acid metabolism (“valine, leucine, and isoleucine degradation” “phenylalanine metabolism” “lysine degradation”) (Figure 6C and Supplementary Table 4). Overall, the microbial communities presented in these two groups could be distinguished based on their functions.

## Discussion

Hippocrates, the father of modern medicine, postulated that “all diseases begin in the gut” (Karlsson et al., 2013; Liang et al., 2019). The human microbiome is an extremely complex ecosystem considering the number of bacterial species, their interactions, and their variability over space and time (Cho and Blaser, 2012). Accumulating evidence documented that the composition and function of gut microbiota are associated with disorders in organ systems in addition to the gastrointestinal system. Recent advances have identified that the gut microbiota plays critical roles in both stimulating and regulating disease progression in ocular diseases, such as autoimmune uveitis (Kalyana Chakravarthy et al., 2018), age-related macular degeneration (AMD) (Zinkernagel et al., 2017), and glaucoma (Gong et al., 2020). However, only a few studies have investigated whether gut microbiota composition changes in DR patients. In the current study, we comprehensively delineated the composition and community structure of gut microbiota in DR patients and healthy individuals using 16S rRNA amplicon sequencing. The comparison of the qualitative and quantitative differences in the composition, abundance, and function of the intestinal flora of these two groups allowed us to determine whether gut flora and their functions contribute to the onset and development of DR.

Microbial community diversity analysis is essential to quantify the bacterial composition and relative abundance of a specific community, including alpha diversity (within communities) and beta diversity (inter-community). In this study, we found that patients in the DR group had distinct gut microbiota compared to the HC group based on the increased microbial richness and altered microbial composition. In addition to the higher sobs indices in DR than in HC, other alpha diversity indices, including Ace, Chao, Shannon, Heip, and Shannon, did not reach statistical difference, although an upregulated trend was observed in Ace and Shannon indexes. Also, the DR group showed significant alterations in beta diversity (PCoA and PLS-DA) compared to the HC group, indicating an obvious distinction between the microbiota composition of these two groups. This finding was consistent with previous studies, wherein the association between Firmicutes and Bacteroidetes had been reported in diabetic patients (Larsen et al., 2010), and the alterations of the composition and function of gut microbiota in metabolic diseases, especially T2DM, were confirmed (Qin et al., 2012; Karlsson et al., 2013). Some studies reported lower alpha diversity indices (Shannon and Chao) of gut microbiota in diabetic patients compared to healthy individuals but different beta diversity, which was not consistent with our results (Nuli et al., 2019; Zhao et al., 2019; Reitmeier et al., 2020). Another study reported an association between bacterial richness and adiposity, insulin resistance, dyslipidemia, and inflammation (Le Chatelier et al., 2013). Therefore, we hypothesized that the underlying mechanism might be the effect of drugs on diabetes.

We observed the community structure of the gut microbiome in DR patients utilizing high-throughput gene sequencing. DR was characterized by alterations in specific OTUs assigned to the families, Lachnospiraceae, Bacteroidaceae, Selenomonadaceae, and Peptostreptococcaceae. A total of 45 genera in DR compared to only 20 in HC were obtained. Thus, we speculated that the microbiota in DR might exhibit a complex and high pathology diversity, proposing that the onset and progression of diabetes and its retinal complications are related to the alterations in gut microbiota.

Furthermore, the alterations in the microbiome of DR patients were related to disturbances in the phyla Firmicutes, Bacteroidetes, Synergistota, and Desulfobacterota. Our results were consistent with those of a previous study on diabetes, which showed a significant reduction in Firmicutes and Clostridia (members of the phylum Firmicutes) and an abundance of Bacteroidetes in the gut of patients with T2DM compared to non-diabetic individuals (Larsen et al., 2010). Similarly, a low abundance of Firmicutes and a high abundance of Bacteroidetes was observed in the DR group, which was consistent with previous findings (Moubayed et al., 2019). The study demonstrated the ratio of Firmicutes to Bacteroidetes (F/B) was significantly and negatively associated with reduced glucose tolerance. Our results also found a significant decline in the F/B ratio in adults with DR. The ratio of these two most important bacterial phyla in the gastrointestinal tract is widely accepted to have an important impact in maintaining normal intestinal homeostasis. As an indicator of gut dysbiosis, the F/B ratio might have an impact on the effectiveness of the gastrointestinal process on the indigestible complex polysaccharides. Restoring the F/B ratio with the proper probiotics can reduce weight gain or suppress the immune system (Turnbaugh et al., 2006).

Gut dysbiosis is a multifactorial disease. In healthy individuals, the intestinal wall prevents the migration of microorganisms and their metabolites from the intestinal lumen to blood circulation. However, gut dysbiosis may relieve the barrier effect of the intestinal lining and cause the leaky gut syndrome. Bacteroidetes are Gram-negative bacteria, whose cell wall is mainly composed of lipopolysaccharide (LPS) and associated with the pathogenesis of diabetes. Bacteroidetes release bacterial endotoxin and LPSs (Davis-Richardson et al., 2014; Vatanen et al., 2016), and trigger innate or natural immunity leading to pro-inflammatory response, which in turn causes vascular dysfunction. A previous study demonstrated that systemic exposure to LPS in hyperglycemic mice could accelerate the process of retinal capillary endothelial cell damage and retinal thinning (Vagaja et al., 2013). We found enriched gut bacteria abundance of Desulfobacterota and Synergistota in the DR group compared to healthy individuals. These bacteria might aggravate abnormalities of energy metabolism. The Desulfobacterota phylum consists of various organisms capable of reducing sulfur compounds via the DsrAB-dissimilatory sulfite reduction pathway, followed by butyrate degradation via the butyrate beta-oxidation pathway (Hao et al., 2020). Hence, we speculated that the disproportion of the F/B ratio and other changes in the intestinal milieu of DR patients could be involved in chronic inflammation and retinal ischemia. Specifically, Bacteroidetes, Desulfobacterota, and Synergistota phyla can either participate in the equilibrium of the catabolic reaction or release LPS to trigger inflammatory injuries; both the above pathological features might cause DR. Considering the significance of previous studies on F/B ratio in diabetes, we will explore its correlation with DR in clinical setting and basic research in future studies.

At the genus level, decreased *Blautia*, *Eubacterium_hallii*_group, *Collinsella*, *Dorea*, *Romboutsia*, *Anaerostipes*, and *Fusicatenibacter* and increased *Bacteroides*, *Megamonas*, *Ruminococcus_torques*_group, *Lachnoclostridium*, and *Alistipes* were observed in the DR group. However, these results were not consistent with those reported in other studies on diabetic patients without retinopathy (Candela et al., 2016; Salamon et al., 2018). Among the commonly reported findings, the genera of *Ruminococcus*, *Lachnoclostridium*, *Dorea*, and *Blautia* were positively associated with diabetes, while the genus of *Bacteroides* was negatively associated with diabetes (Zhang et al., 2013; Candela et al., 2016; Inoue et al., 2017; Lippert et al., 2017; Gurung et al., 2020; Wang et al., 2020). The alterations of *Blautia* and *Bacteroides* genera are partially contradictory to the expectations, which could be attributed to the effect of drugs for the treatment of diabetes treatment and compensatory growth of bacteria. The critical effects of some drugs for diabetes treatment on gut microbiome alterations have been confirmed in some recent studies; for example, Wu et al. (2017) showed that metformin had critical effects on gut microbiome (including increased *Bifidobacterium*). Most DR patients in the current study used metformin and other drugs for their treatment for many years. Another possible reason is the existence of gut-retina axis, which might lead to slight but critical change in the intestinal microecology of diabetic patients with or without retinopathy. Huang et al. (2021) reported that *Lactobacillus* genus was enriched in participants with diabetes and was more abundant in the type 2 diabetic patients than in the DR patients. However, no studies have yet explored these potentially causal bacteria in animal models of diabetes or DR; thus, the effects of these bacteria on DR require further study in basic research setting and clinical research with large sample sizes.

In this study, we found that individuals with DR have decreased abundance of several bacteria with the potential to produce butyrate. For example, *Eubacterium*_*hallii*_group (an anaerobic, Gram-positive, catalase-negative bacterium of the Lachnospiraceae family), is capable of butyrate production (Karlsson et al., 2013; Hiippala et al., 2018). In our study, decreased *Eubacterium_hallii*_group were observed in the DR group, which was concordant with those reported previously, they found decreased abundance of butyrate-producing bacteria in patients with diabetes (Qin et al., 2012; Karlsson et al., 2013; Forslund et al., 2015). *Eubacterium_hallii*_group ferment dietary fibers to metabolize butyric acid and propionate through intestinal microbes, thereby regulating insulin sensitivity, reducing inflammation, and improving diabetes in humans and mice (Schwab et al., 2017; Shetty et al., 2017). In addition, oral treatment with the *Eubacterium_hallii_*group improved insulin sensitivity in db/db mice (Udayappan et al., 2016), indicating that the bacteria are highly discriminant in DR patients. *Collinsella*, one of the most abundant Actinobacteria, in the intestinal flora of healthy people, is a known human gut colonizer and one of its subspecies is a butyrate producer (Qin et al., 2019). Hence, we speculated that the occurrence of diabetes and retinopathy might be associated with a reduction in these beneficial bacteria. *Dorea* is a new member of *Clostridium* cluster XIVa in the Lachnospiraceae family, which could produce acetate as a fermentation product (Brahe et al., 2015). Moreover, decreased *Dorea* abundance has been associated with both positive and negative conditions, but there is no consensus on its effects on eye disease (Allin et al., 2018; Li et al., 2020).

In this study, we found that the abundance of *Megamonas* and *Alistipes* was elevated in the DR group. Shang et al. (2021) showed that *Megamonas* was significantly correlated with DR, duration of diabetes, and older age. Zhu et al. (2020) noted that the abundance of *Alistipes* was significantly increased in T2DM rats. In addition, we identified some microbiota, including *Romboutsia*, *Anaerostipes*, and *Fusicatenibacter* genera, which were further investigated the detailed mechanism of the disturbance of these microbiotas in the onset and development of DR, although they have not been previously reported in gut microbiota on diabetes or DR. Thus, our findings indicated that these abnormally abundant gut bacteria might play a critical and complex role in metabolic and ocular diseases. Therefore, additional studies are needed to substantiate these findings.

In addition to identifying the distinct gut microbiota from the phylum to the genus level in each group, we established a model that can distinguish subjects with DR from the HC group with a reliable diagnostic accuracy using the random forest prediction model. We selected and confirmed 7 microbial biomarkers at the genus level with the highest AUC of 0.97 based on the microbial characteristics. These gut microbiomes could be used as novel targets for clinical non-invasive diagnostic biomarkers and therapeutic interventions for DR in the future. However, dysbiosis of the gut microbiome varies from person to person; thus, our results need to be confirmed in clinical trials. Furthermore, we analyzed the functional and metabolic changes in the microbial communities between the two groups. Notably, the pathways involved in “retinol metabolism” were increased, and those involved in “insulin resistance” were suppressed in the DR group, providing valuable information for investigating the role of microbiota in the gut-retina axis, although these predicted pathways were not confirmed experimentally.

Clinical value of gut microbiota in ocular diseases need to be further confirmed in the future. The future treatment of ocular disorders might be strictly linked to gut microbiota. Increased understanding of the link of gut microbiota and ocular diseases will provide potential new therapeutic strategies targeting commensal microbiota. For example, dietary intervention including probiotic and relative metabolite regimens had been proposed as potential therapy in recent years.

The major merit of this study was that we used the strict and ingenious control-matching method to recruit the subjects. Previous studies have demonstrated that microbiota composition is affected by several factors, including genetics, drugs, and diet (Ianiro et al., 2016; Hills et al., 2019). In this study, we recruited the spouses of patients with DR as healthy controls. We presumed that their nutrition was similar based on the same dietary structures. On the other hand, individuals who used probiotics, prebiotics, or antibiotics recently were excluded to reduce the bias resulting from potential confounding effects, including diet, age, BMI, and medications. Nevertheless, the study had the following limitations. Firstly, 16S rRNA gene sequencing was deficient in species level and functional analysis compared to metagenome sequencing. Secondly, this study was a cross-sectional study, and hence, a causal link between the differences in gut microbiome composition identified in this study could not be established. Future multicenter and large cohort studies can reduce potential bias, which may help to further verify the results of this study. Furthermore, it is difficult to interpret whether the microbial dysbiosis that exists in diabetic patients with or without retinopathy due to the lack of direct comparison between DR patients and diabetic patients without retinopathy. Despite these limitations, the findings of this study provided in-depth insights into the role of gut microbiota in the pathogenesis of diabetes and its ocular complications. In the future, detailed and comprehensive investigations of the sequential changes in gut microbiota in DR patients could be carried out in multicenter controlled trials and comprehensive and complete experiments. Also, animal experiments might provide a molecular mechanism and determine the cause-effect correlation between the pathogenesis of DR and bacteria.

## Conclusion

The gut microbiome plays crucial roles in normal development and homeostasis in humans, which also affects eye health. Accumulating studies have shown that the progression of eye disease is related to alterations in the composition and function of gut microbes. In this study, we characterized and identified the composition and diversity of fecal microbiota in DR patients and compared them to healthy individuals using high-throughput gene sequencing techniques. Alterations of gut microbial composition, structure, and function were observed at different taxonomic levels in DR patients, suggesting the critical role of metabolic activities of microbiota in the pathogenesis and progression of DR. Thus, comprehensive characterization of the DR-associated fecal microbiota will lay the foundation for clinical diagnostic research and identification of potential therapeutics in the future.

## Data availability statement

The datasets presented in this study can be found in online repositories. The names of the repository/repositories and accession number(s) can be found below: NCBI, PRJNA857030.

## Ethics statement

The studies involving human participants were reviewed and approved by the Medical Ethics Committee of the Shanghai Tenth People’s Hospital of Tongji University. The patients/participants provided their written informed consent to participate in this study. Written informed consent was obtained from the individual(s) for the publication of any potentially identifiable images or data included in this article.

## Author contributions

JB and ZW participated in the data analysis and writing of the manuscript. TW, YZ, and YX participated in data collection and revising the manuscript. QP designed the study and wrote and revised the manuscript. All authors contributed to the article and approved the submitted version.

## Footnotes

1
http://www.chictr.org.cn

2
http://drive5.com/uparse/

## Supplementary material

Supplementary Table 1 The sequencing results of each sample.

Supplementary Table 2 A total of 6507 Kyoto Encyclopedia of Genes and Genomes (KEGG) ortholog (KO) genes were predicted.

Supplementary Table 3 A total of 335 KEGG pathways were predicted.

Supplementary Table 4 The significantly differential pathway at level 3 between DR patients and HC individuals.
